# Supplementary material for: Coumarins from Seseli devenyense Simonk.: Isolation by Liquid–Liquid Chromatography and Potential Anxiolytic Activity Using an In Vivo Zebrafish Larvae Model
Source: Int J Mol Sci. 2021 Feb 12;22(4):1829. doi: 10.3390/ijms22041829 (PMC7918798; doi:10.3390/ijms22041829)
Supplement: Supplementary file 1 [file ijms-22-01829-s001.pdf]

Supplementary Materials

Coumarins from *Seseli devenyense* Simonk.: Isolation by liquid-liquid chromatography and potential anxiolytic activity using an *in vivo* zebrafish larvae model

Jarosław Widelski [1,] * Simon Vlad Luca [2,3], Adrianna Skiba [4], Monika Maciąg [5,6], Barbara Budzyńska [5], Laurence Marcourt [7], Jean-Luc Wolfender [7] and Krystyna Skalicka-Woźniak [4]

1 Department of Pharmacognosy with Medicinal Plant Unit, Medical University of Lublin, 20-093 Lublin, Poland; jwidelski@pharmacognosy.org (J.W)
2 Biothermodynamics, TUM School of Life Sciences, Technical University of Munich, 85354 Freising, Germany; vlad.luca@tum.de (S.V.L)
3 Department of Pharmacognosy, *Grigore T. Popa* University of Medicine and Pharmacy Iasi, 700115 Iasi, Romania
4 Independent Laboratory of Natural Products Chemistry, Medical University of Lublin, 20-093 Lublin, Poland; adrianna.skiba@umlub.pl (A.S); kskalicka@pharmacognosy.org (K.S.-W.)
5 Independent Laboratory of Behavioral Studies, Medical University of Lublin, 20-093 Lublin, Poland; monika.maciag@umlub.pl (M.M); barbara.budzynska@umlub.pl (B.B)
6 Department of Biopharmacy, Medical University of Lublin, 20-093 Lublin, Poland
7 School of Pharmaceutical Sciences, Institute of Pharmaceutical Sciences of Western Switzerland, IPSWS, University of Geneva, CMU, 1211 Geneva 4, Switzerland; laurence.marcourt@unige.ch (L.M.); Jean-Luc.Wolfender@unige.ch (J.-L.W)
* Correspondence: jwidelski@pharmacognosy.org

Supplementary File Content

Part A. Chromatograms of: *Seseli devenyense* Simonk. methanolic fruit extract, on-line chromatogram of the CCC separations of the target coumarins from *Seseli devenyense* methanolic fruit extract and isolated compounds

Part B. Spectroscopic data of isolated compounds

Part C. The influence of *Seseli devenyense* methanolic fruit extract (SDMFE) and isolated coumarins on spontaneous locomotor activity and thigmotaxis behaviour of the zebrafish larvae

C1. The influence of SDMFE on spontaneous locomotor activity and thigmotaxis behaviour of the zebrafish larvae

C2. The influence of devenyol on spontaneous locomotor activity and thigmotaxis behaviour of the zebrafish larvae

C3. The influence of cis-khellactone on spontaneous locomotor activity and thigmotaxis behaviour of the zebrafish larvae

C4. The influence of d-laserpitin on spontaneous locomotor activity and thigmotaxis behaviour of the zebrafish larvae

C5. The influence of isolaserpitin on spontaneous locomotor activity and thigmotaxis behaviour of the zebrafish larvae

C6. The influence of octanoyllomatin on spontaneous locomotor activity and thigmotaxis behaviour of the zebrafish larvae

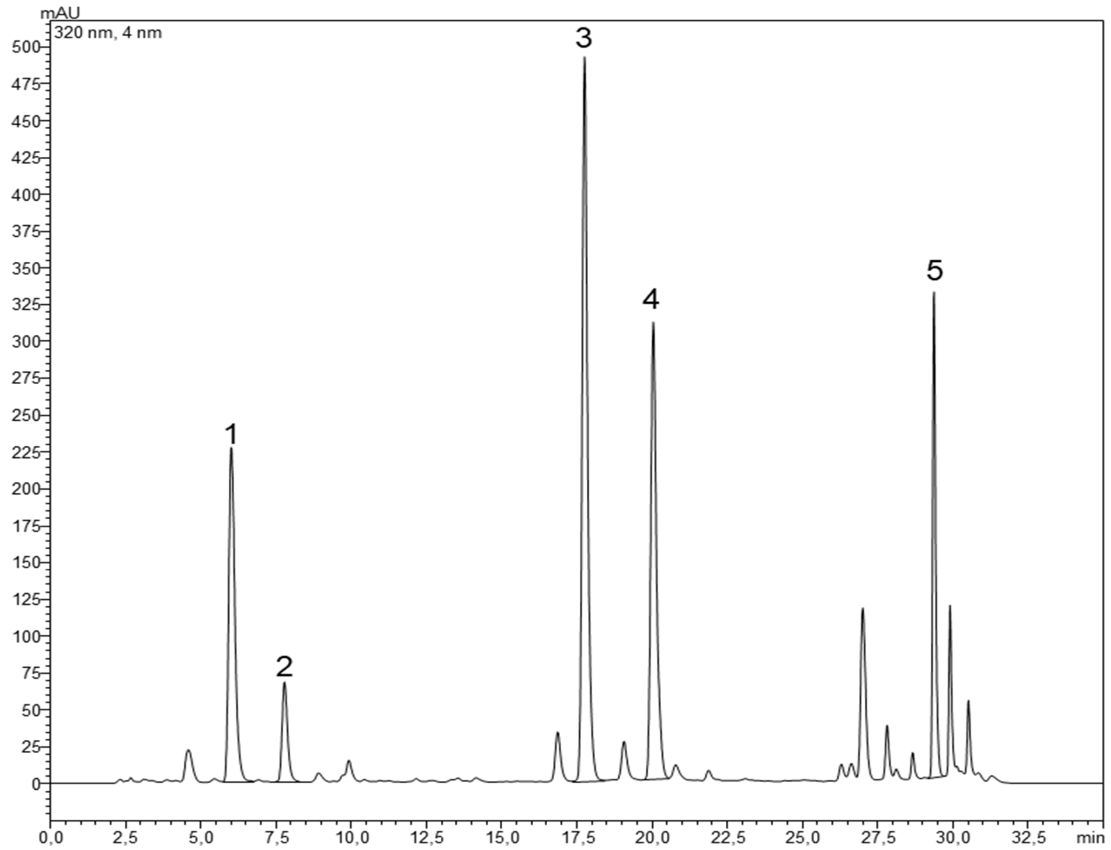

Figure S1. HPLC-DAD chromatogram of *Seseli devenyense* Simonk. methanolic fruit extract (320 nm); coumarin **1** (T_R=6.0 min), coumarin **2** (T_R=7.7 min), coumarin **3** (T_R =17.7 min), coumarin **4** (T_R =20.0 min) and coumarin **5** (T_R =29.3 min).

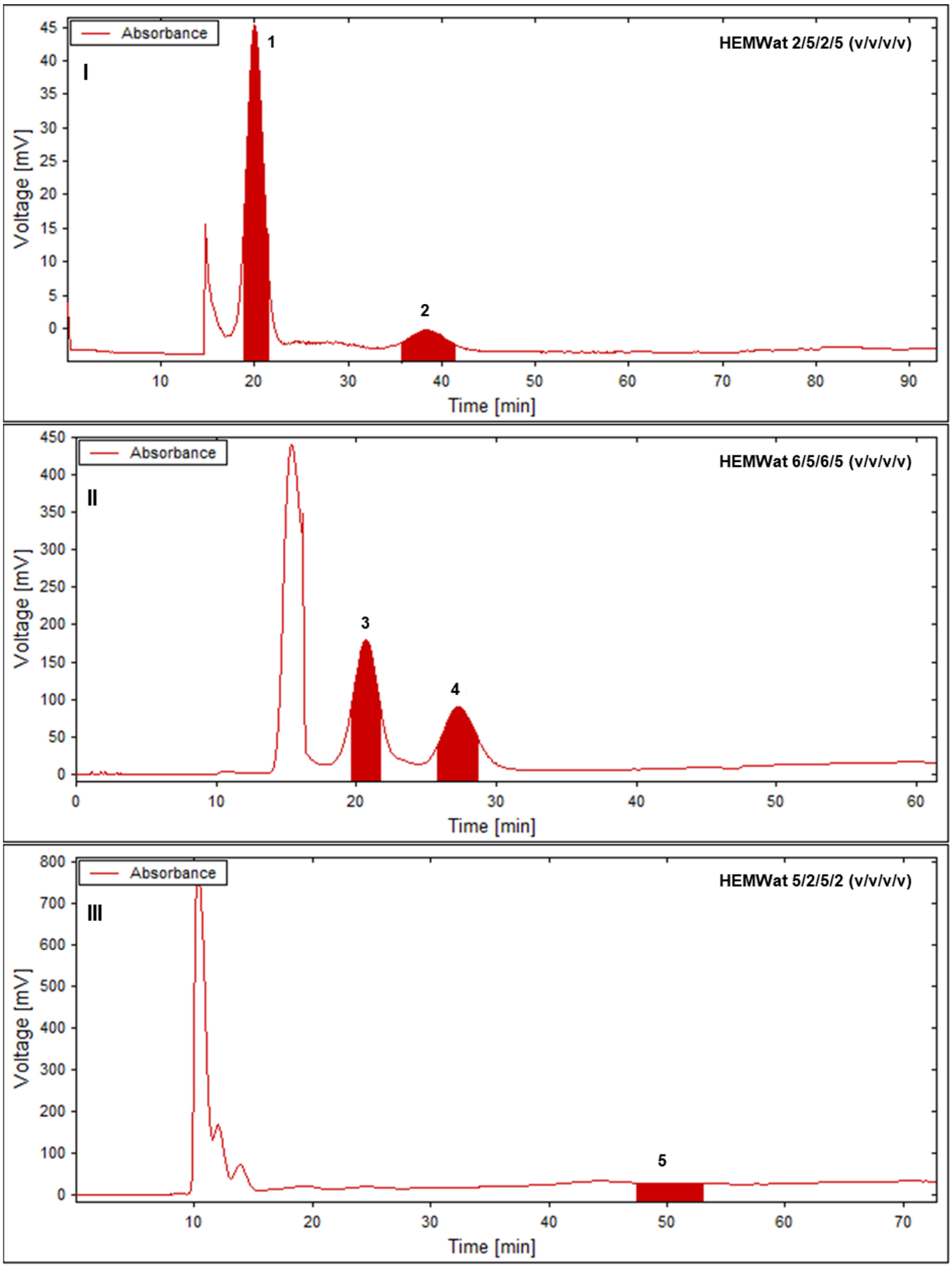

Figure S2. On-line chromatogram of the CCC separations of the target coumarins from *Seseli devenyense* methanolic fruit extract**.** Unit: CCC (Dynamic Extractions, Slough, UK) with the column volume: 136 mL; Elution mode: reversed-phase mode (lower phase as mobile phase, head-to-tail, descending mode); Flow-rate: 6 mL/min; Rotation speed: 1600 rpm; Injection volume: 6 mL; Extract concentration: 40 mg/mL; UV: 320 nm; *Separation I* with hexane/ethyl acetate/methanol/water (2/5/2/5, v/v/v/v); *Separation II* with hexane/ethyl acetate/methanol/water (6/5/6/5, v/v/v/v); *Separation III* with hexane/ethyl acetate/methanol/water (5/2/5/2, v/v/v/v).

Part A. Chromatograms of isolated compounds

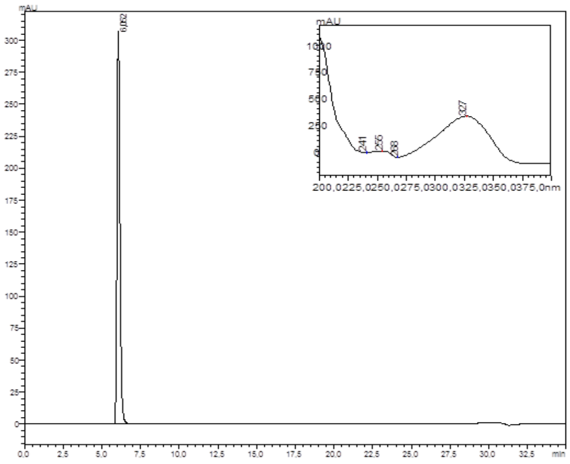

(**1**)

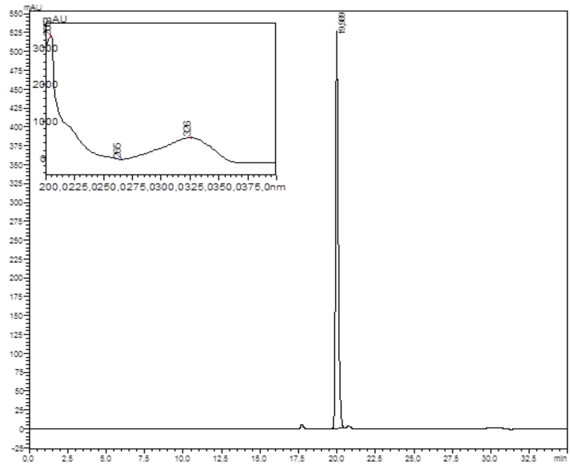

(**4**)

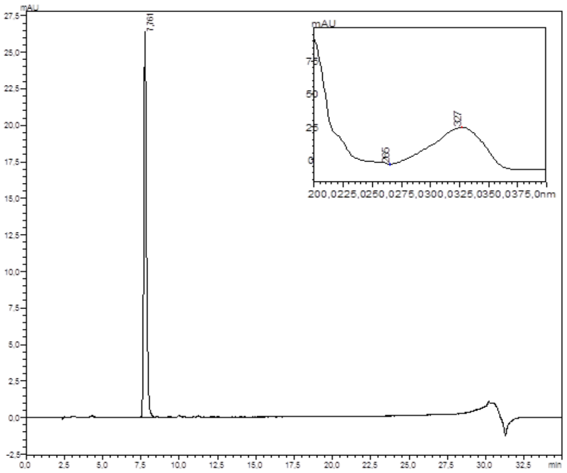

(**2**)

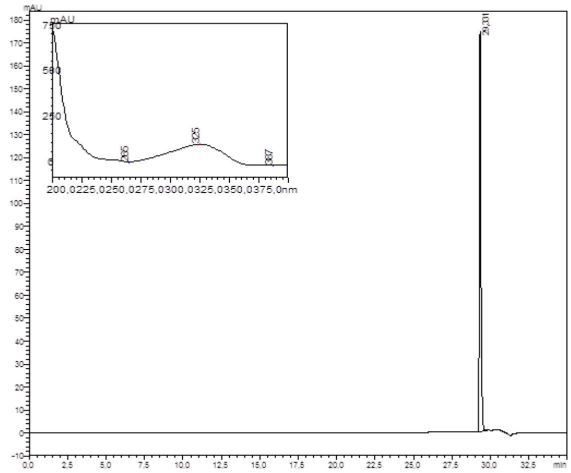

(**5**)

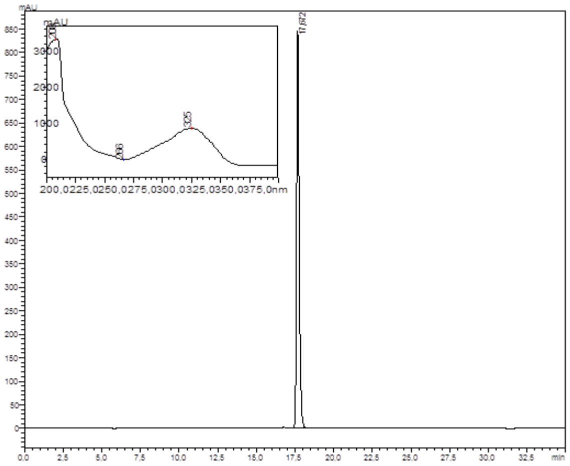

(**3**)

Figure S3. HPLC-DAD chromatograms of devenyol (**1**), *cis*-kellactone (**2**), d-laserpitin (**3**), isolaserpitin (**4**) and octanoyllomatin (**5**)

Part B. Spectroscopic data of isolated compounds

*Devenyol (**1**):* UV (methanol) λ_{max} 327 nm; ^{1}H NMR (CD_3OD, 600 MHz) δ_H 7.90 (1H, d, *J*=9.4 Hz, H-4), 7.36 (1H, d, *J*=8.4 Hz, H-5), 6.80 (d, *J*=8.4 Hz, H-6), 6.15 (d, *J*=9.4 Hz, H-3), 3.52 (1H, dd, *J*=10.1, 2.6 Hz, H-2'), 2.92 (1H, dd, *J*=13.5, 2.6 Hz, H-1a'), 2.77 (1H, dd, *J*=13.5, 10.1 Hz, H-1b'), 1.14 (6H, s, H-4', H-5'); ^{13}C NMR (CD_3OD, 151 MHz) δ_C 160.7 (C-2), 153.9 (C-9), 145.0 (C-4), 126.7 (C-5), 114.4 (C-8), 112.7 (C-6), 111.1 (C-10), 110.5 (C-3), 76.9 (C-2'), 71.9 (C-3'), 25.8 (C-4'), 25.3 (C-5'), 25.1 (C-1'); HREIMS *m/z* 287.0897 [M+Na]+ (calcd for $C_{14}H_{16}O_5Na$ 287.0890, Δ = –2.12 ppm); MS/MS (10 eV) *m/z* 269.0818, 247.0924, 229.0800, 175.0374, 131.0510.

*cis-Khellactone (**2**):* UV (methanol) λ_{max} 327 nm; ^{1}H NMR (CD_3OD, 600 MHz) δ_H 7.97 (1H, d, *J*=9.4 Hz, H-4), 7.52 (1H, d, *J*=8.6 Hz, H-5), 6.76 (d, *J*=8.6 Hz, H-6), 6.28 (d, *J*=9.4 Hz, H-3), 5.21 (1H, d, *J*=4.6 Hz, OH-1'), 5.14 (1H, d, *J*=6.6 Hz, OH-2'), 4.91 (1H, t, *J*=4.6 Hz, H-1'), 3.62 (1H, dd, *J*=6.6, 4.6 Hz, H-2'), 1.36 (6H, s, H-4', H-5'); ^{13}C NMR (CD_3OD, 151 MHz). HREIMS *m/z* 263.0916 [M+H]+ (calcd for $C_{14}H_{15}O_5$ 263.0914, Δ = –0.76 ppm); MS/MS (10 eV) *m/z* 245.0827, 215.0748, 203.0715, 187.0350, 175.0387, 147.0465, 131.0559, 107.0462.

*d-Laserpitin (**3**):* UV (methanol) λ_{max} 325 nm; ^{1}H NMR (CD_3OD, 600 MHz) δ_H 7.97 (1H, d, *J*=9.5 Hz, H-4), 7.60 (1H, d, *J*=8.6 Hz, H-5), 6.83 (d, *J*=8.6 Hz, H-6), 6.39 (1H, d, *J*=5.1 Hz, H-1'), 6.27 (d, *J*=9.4 Hz, H-3), 5.98 (1H, qd, *J*=7.2, 1.6 Hz, H-3''), 5.69 (1H, d, *J*=6.3 Hz, OH-2'), 3.94 (1H, dd, *J*=6.3, 5.0 Hz, H-2'), 1.88 (3H, dq, *J*=7.2, 1.5 Hz, H-4''), 1.79 (3H, p, *J*=1.5 Hz, H-5''), 1.39 (3H, s, H-4'), 1.32 (3H, s, H-5'); ^{13}C NMR (CD_3OD, 151 MHz) δ_C 166.2 (C-1''), 159.5 (C-2), 156.4 (C-7), 153.7 (C-9), 144.5 (C-4), 135.6 (C-5), 128.1 (C-2''), 114.1 (C-6), 112.2 (C-3), 112.0 (C-10), 108.1 (C-8), 78.7 (C-3'), 69.1 (C-2'), 61.7 (C-1'), 26.1 (C-4'), 20.5 (C-5'), 20.1 (C-5''), 15.2 (C-4''); HREIMS *m/z* 345.1346 [M+H]+ (calcd for $C_{19}H_{21}O_6$ 345.1333, Δ = –3.88 ppm); MS/MS (10 eV) *m/z* 327.1310, 245.0866, 227.0756, 215.0727, 203.0762, 175.0435.

*Isolaserpitin (**4**):* UV (methanol) λ_{max} 326 nm; ^{1}H NMR (CD_3OD, 600 MHz) δ_H 7.99 (1H, d, *J*=9.5 Hz, H-4), 7.58 (1H, d, *J*=8.6 Hz, H-5), 6.82 (d, *J*=8.6 Hz, H-6), 6.31 (1H, d, *J*=9.5 Hz, H-3), 6.20 (dddd, *J*=8.7, 7.2, 5.8, 1.5 Hz, H-3''), 5.77 (1H, d, *J*=5.7 Hz, OH-1'), 5.23 (1H, dd, *J*=5.7, 4.5 Hz, H-1'), 4.92 (1H, d, *J*=4.5 Hz, H-2'), 1.97 (3H, dt, *J*=7.2, 1.5 Hz, H-4''), 1.90 (3H, m, H-5''), 1.45 (3H, s, H-4'), 1.38 (3H, s, H-5'); ^{13}C NMR (CD_3OD, 151 MHz) δ_C 166.3 (C-1''), 160.1 (C-2), 155.3 (C-7), 153.8 (C-9), 144.6 (C-4), 138.5 (C-5), 127.3 (C-2''), 113.9 (C-6), 112.3 (C-3), 112.2 (C-10), 111.1 (C-8), 77.0 (C-3'), 73.6 (C-2'), 57.7 (C-1'), 26.3 (C-4'), 22.2 (C-5'), 20.3 (C-5''), 15.6 (C-4'').

*Octanoyllomatin (**5**):* UV (methanol) λ_{max} 325 nm; ^{1}H NMR (CD_3OD, 600 MHz) δ_H 7.98 (1H, d, *J*=9.5 Hz, H-4), 7.49 (1H, d, *J*=8.6 Hz, H-5), 6.83 (d, *J*=8.6 Hz, H-6), 6.28 (1H, d, *J*=9.5 Hz, H-3), 5.10 (1H, t, *J*=4.1 Hz, H-2'), 3.13 (1H, dd, *J*=18.0, 4.7 Hz, H-1a'), 2.87 (1H, dd, *J*=18.0, 3.5 Hz, H-1b'), 2.27 (1H, t, *J*=7.1 Hz, H-2''), 1.45 (1H, p, *J*=6.8 Hz, H-3''), 1.34 (3H, s, H-4'), 1.29 (3H, s, H-5'), 1.17 (2H, m, H-7'') 1.12 (6H, m, H-4'', H-5'', H-6''), 0.80 (6H, t, *J*=7.2 Hz, H-8''); ^{13}C NMR (CD_3OD, 151 MHz) δ_C 172.4 (C-1''), 160.1 (C-2), 155.7 (C-7), 152.9 (C-9), 144.7 (C-4), 127.2 (C-5), 113.7 (C-6), 112.0 (C-3), 111.9 (C-10), 106.1 (C-8), 76.4 (C-3'), 68.5 (C-2'), 33.5 (C-2''), 31.0 (C-6''), 28.3 (C-4''), 24.5 (C-3''), 23.9 (C-5'), 23.4 (C-4'), 22.3 (C-1'), 21.9 (C-7''), 13.9 (C-8''); HREIMS *m/z* 373.2026 [M+H]+ (calcd for $C_{22}H_{29}O_5$ 373.2010, Δ = –4.43 ppm); MS/MS (10 eV) *m/z* 247.0942, 229.0851, 201.0864, 187.0408, 159.0434, 131.0453.

Part C. The influence of *Seseli devenyense* methanolic fruit extract and isolated coumarins on spontaneous locomotor activity and thigmotaxis behaviour of the zebrafish larvae

C1. The influence of SDMFE on spontaneous locomotor activity and thigmotaxis behaviour of the zebrafish larvae

The spontaneous locomotor activity was measured as average distance (in mm) moved during 40 minutes of continuous illumination. One-way ANOVA [$F(6,224)=9.917$; $p<0.0001$] revealed that SDMFE fruits influenced on the spontaneous locomotor activity. Post-hoc Tukey's test confirmed that extract at the concentrations 3 μg/mL ($p<0.01$), 6 μg/mL ($p<0.001$), 9 μg/mL ($p<0.01$) and 12.5 μg/mL ($p<0.05$) increased spontaneous locomotor activity of *Danio rerio* larvae comparing to control group **(Figure S4a)**.

One-way ANOVA showed statistically significant differences between groups concerning distance moved in the central arena during 40 min [$F(6, 224)=8.820$, $p<0.0001$]. Post-hoc Tukey's test confirmed that diazepam ($p<0.05$) and SDMFE at the concentration of 12.5 μg/mL ($p<0.05$) and 25 μg/mL ($p<0.01$) increased a distance moved in the central arena (**Figure S4b)**.

One-way ANOVA [$F(6,224)=10.90$, $p<0.0001$] showed, that time spent in central arena after 30 minutes incubation in different concentrations of SDMFE increase statistically significant in time spent in the central arena. Post-hoc Tukey's test showed that diazepam ($p<0.05$) and SDMFE at the concentration of 25 μg/mL ($p<0.001$) increased the percentage of time spent in the central arena in comparison to the control group, treated with DMSO (**Figure S4c**).

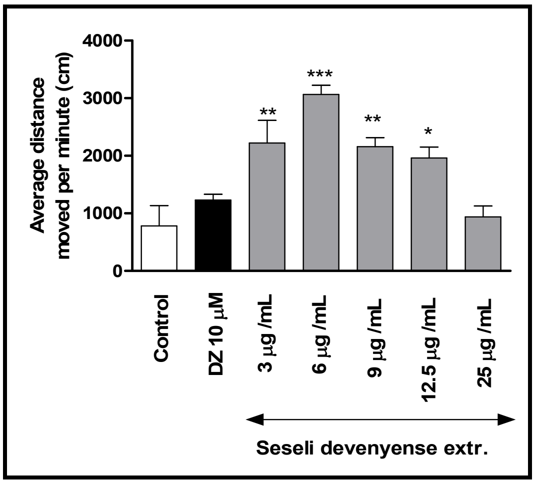

(**a**)

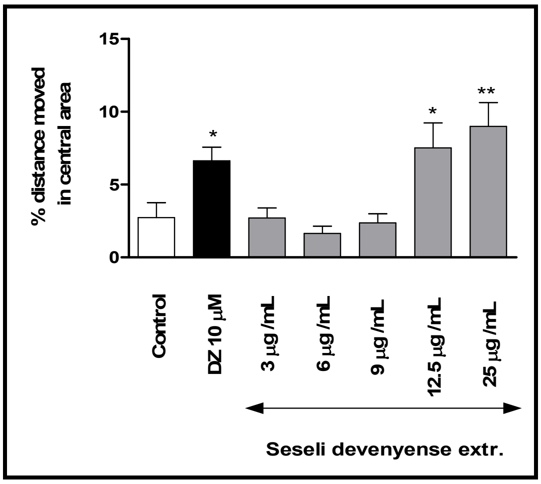

(**b**)

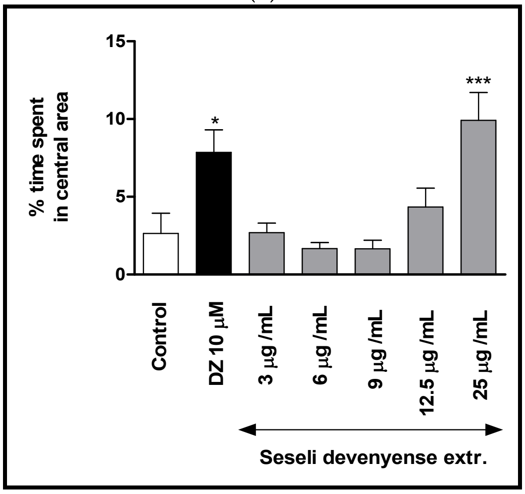

(**c**)

Figure S4. The effects of SDMFE (3, 6, 9, 12.5, 25 μg/mL) and diazepam (10 μM) on locomotor activity during 40 min light phase. (**a**) Average distance (mm) moved by zebrafish larvae within each 1-min time bin; (**b**) The percentage of the distance moved in the central arena under continuous illumination;

(**c**) the percentage of the time spent in the central arena under continuous illumination. Data are presented as mean±SEM, n=32. *p<0.05, **p<0.01, ***p<0.001 in comparison to control group, post hoc Tukey's test

C2. The influence of devenyol on spontaneous locomotor activity and thigmotaxis behaviour of the zebrafish larvae

The spontaneous locomotor activity was measured as average distance (in mm) moved in a minute during 40 minutes of continuous illumination. One-way ANOVA ($F(6, 220)=0{,}5988$; $p=0.7302$) revealed that devenyol at all concentrations (1.5,3, 6, 9, 15 μM) has not influenced the spontaneous locomotor activity (**Figure S5a**).

Furthermore, one-way ANOVA has shown statistically significant differences between groups concerning distance moved in central arena ($F(6, 220)=5.551$, $p=0.0001$), as well as time spent in central arena ($F(6, 220)=4.395$, $p=0.0009$). Post-hoc Tuckey's test confirmed that diazepam increased the percentage of distance moved ($p<0.01$) and time spent ($p<0.01$) in central arena in comparison to DMSO-treated group (control group) (**Figure S5b,c**).

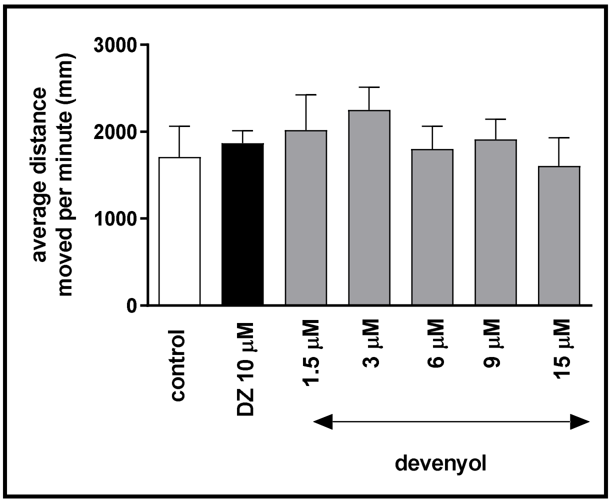

(**a**)

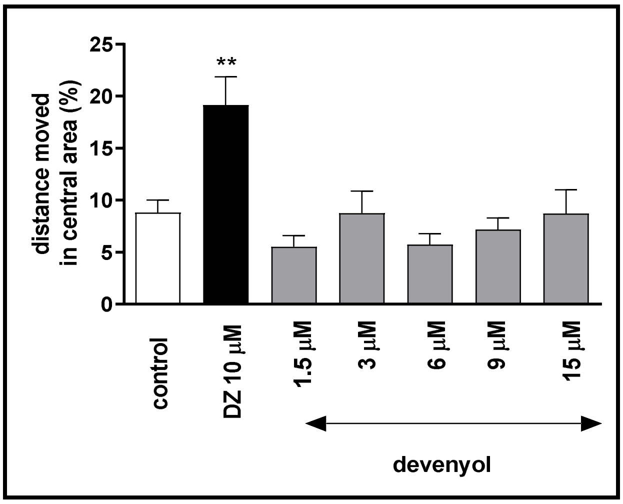

(**b**)

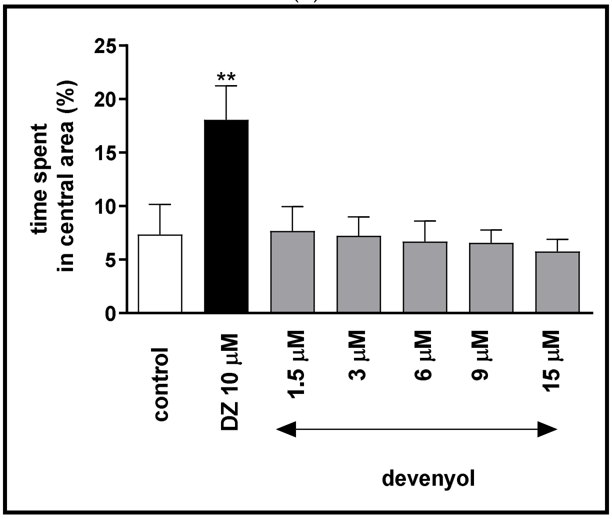

(**c**)

Figure S5. The effect of devenyol (1.5, 3, 6, 9, 15 μM) and diazepam (10 μM) on locomotor activity during 40 min light phase. (**a**) Average distance (mm) moved by zebrafish larvae within each 1-min time; (**b**) The percentage of the distance moved in the central arena under the continuous illumination; (**c**) the percentage of the time spent in the central arena under the continuous illumination. Data are presented as mean ± SEM, n=32, **p<0.01, in comparison to control group, post hoc Tukey's test

C3. The influence of cis-khellactone on spontaneous locomotor activity and thigmotaxis behaviour of the zebrafish larvae

The spontaneous locomotor activity was measured as the average distance (in mm) moved during 40 minutes of continuous illumination. One-way ANOVA [F(7, 256) = 3.365, p=0.3195] revealed that cis-khellactone in all tested concentrations (1.5, 3, 6, 9, 12.5, 25 μM) has not influenced the spontaneous locomotor activity in zebrafish larvae (5-dpf) **(Figure S6a)**.

Furthermore, one-way ANOVA has not shown statistically significant differences between the tested group when distance moved in the central arena during 40 minutes of continuous light was considered [F (7, 256) = 4.478 , p = 0.0011) **(Figure S6b)**.

One-way ANOVA has not showed also statistically significant differences in time spent in the central arena [F (7, 256) = 3.444, p=0.0088] and post-hoc test (Tukey's test) confirmed that diazepam (p<0.05) increase this parameter in comparison to DMSO-treated group (control group) (**Figure S6c**).

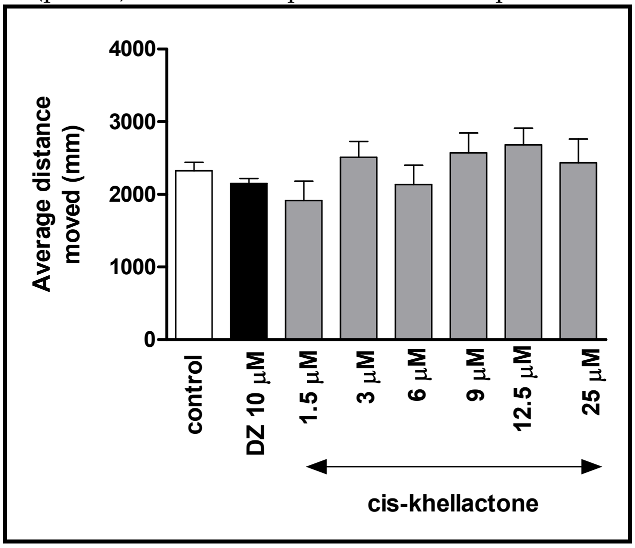

(**a**)

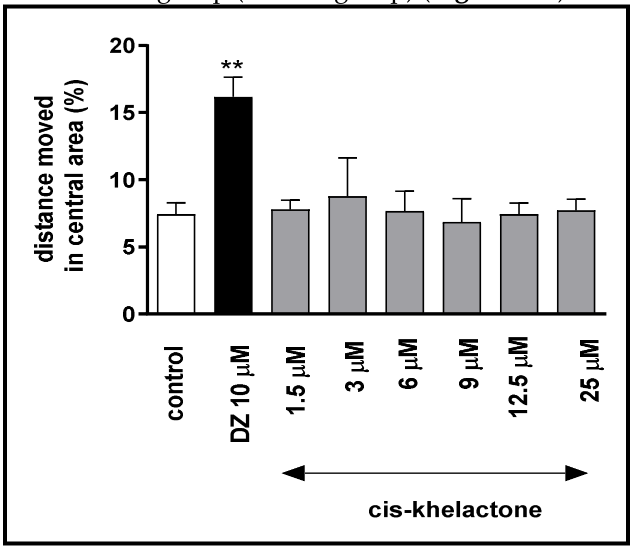

(**b**)

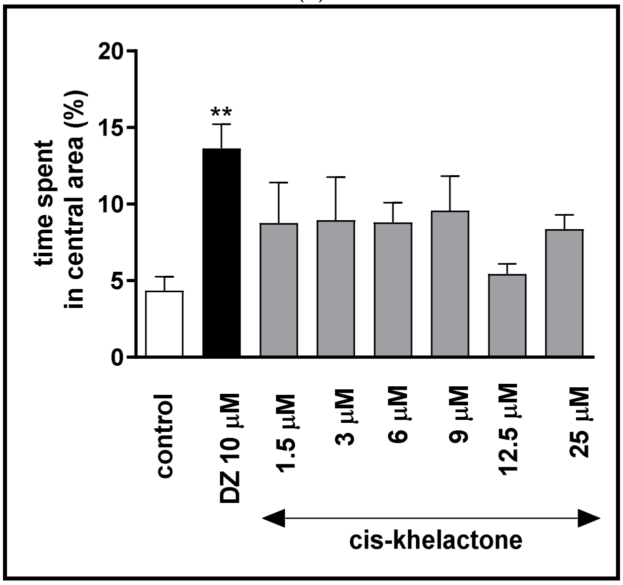

(**c**)

Figure S6 The effect of cis-khellactone (1.5, 3, 6, 9, 12.5, 25 μM) and diazepam (10 μM) on locomotor activity during 40 min light phase. (**a**) Average distance (mm) moved by zebrafish larvae within each 1-min time bin; (**b**) the percentage of the distance moved in the central arena under the continuous illumination; (**c**) the percentage of the time spent in the central arena under the continuous illumination. Data are presented as mean ± SEM, n=32, **p<0.01 in comparison to control group, post hoc Tukey's test

C4. The influence of d-laserpitin on spontaneous locomotor activity and thigmotaxis behaviour of the zebrafish larvae

The spontaneous locomotor activity was measured as the average distance (in mm) moved during 40 minutes of continuous illumination. One-way ANOVA (F (4, 160) = 0.6133; p=0,6582) revealed that d-laserpitin in all concentrations (12.5, 25, 50 µM) has not influenced the spontaneous locomotor activity **(Figure S7a)**.

Furthermore, one-way ANOVA showed statistically significant differences between the tested group when distance moved in the central arena during 40 minutes of continuous light was considered (F (4, 160) = 5.268; p=0.0055) **(Figure S7b)**.

Post-hoc Tukey's test confirmed that diazepam at concentration 10 µM ($p<0.05$) increased the percentage of distance moved in the central arena in comparison with DMSO-treated group **(Figure S7b)**.

One-way ANOVA showed also statistically significant differences in time spent in the central arena [F (4, 160) = 4.734; p=0,0095] and post-hoc test (Tukey's test) confirmed that diazepam ($p<0.05$) increased this parameter in comparison to DMSO-treated group (control group) (**Figure S7c**).

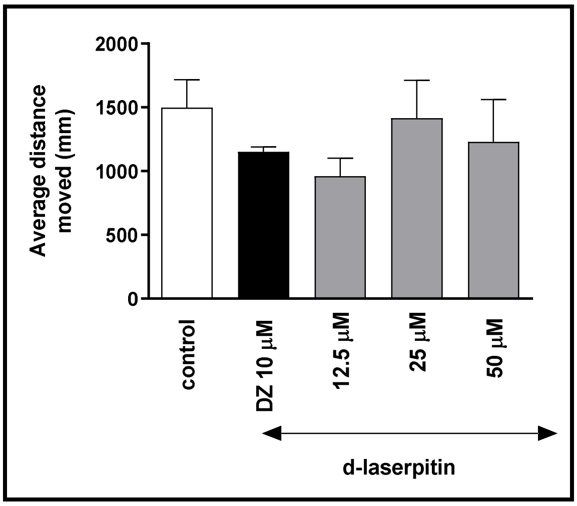

(**a**)

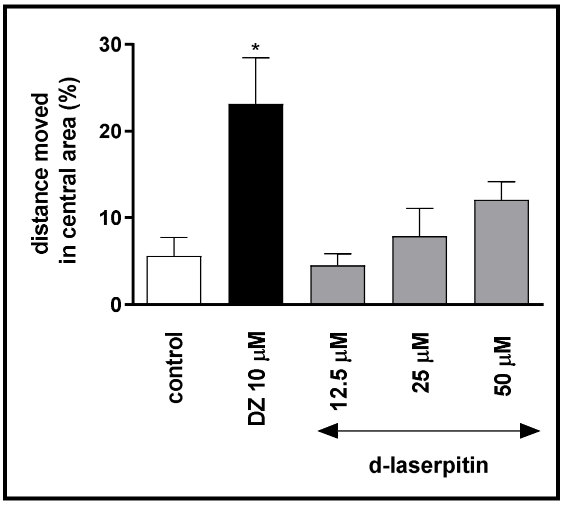

(**b**)

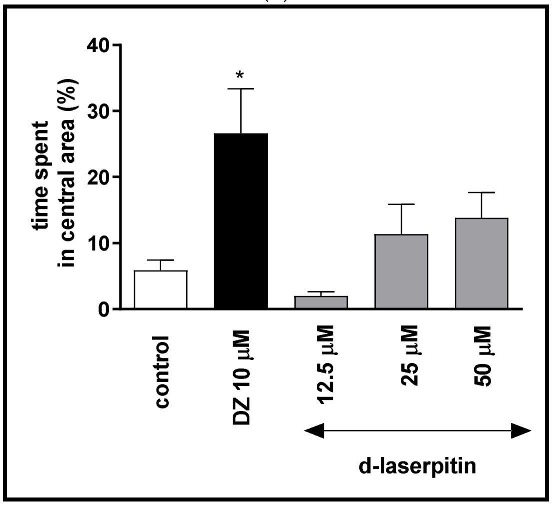

(**c**)

Figure S7. The effect of d-laserpitin (12, 25, 50 µM) and diazepam (10 µM) on locomotor activity during 40 min light phase. (**a**) Average distance (mm) moved by zebrafish larvae within each 1-min time bin; (b) the percentage of the distance moved in the central arena under the continuous illumination. **(c)** the percentage of the time spent in the central arena under the continuous illumination. Data are presented as mean ± SEM, n=32, *p<0.05, in comparison to control group, post hoc Tukey's test.

C5. The influence of isolaserpitin on spontaneous locomotor activity and thigmotaxis behaviour of the zebrafish larvae

The spontaneous locomotor activity was measured as the average distance (in mm) moved during 40 minutes of continuous illumination. One-way ANOVA ($F(4,160)=4.012$; $p=0.159$) revealed that isolaserpitin in all concentrations (12.5,25,50 μM) has not influenced the spontaneous locomotor activity **(Figure S8a)**. Furthermore, one-way ANOVA showed statistically significant differences between the tested group when distance moved in the central arena during 40 minutes of continuous light was considered ($F(4,160)=94.27$, $p<0.0001$) **(Figure S8a**).

Post-hoc Tukey's test confirmed that diazepam ($p<0.001$) and isolaserpitin at concentrations of 12.5 μM ($p<0.001$) and 50 μM ($p<0.05$) increased the percentage of distance moved in the central arena in comparison with DMSO-treated group **Figure S8b**).

One-way ANOVA showed also statistically significant differences in time spent in the central arena [$F(4,160)=76.82$, $p<0.001$] and post-hoc test (Tukey's test) confirmed that as well diazepam ($p<0.05$) and isolasepitin at concentration 12.5 μM/mL(0<0.001) increase this parameter in comparison to DMSO-treated group (control group) (**Figure S8c**).

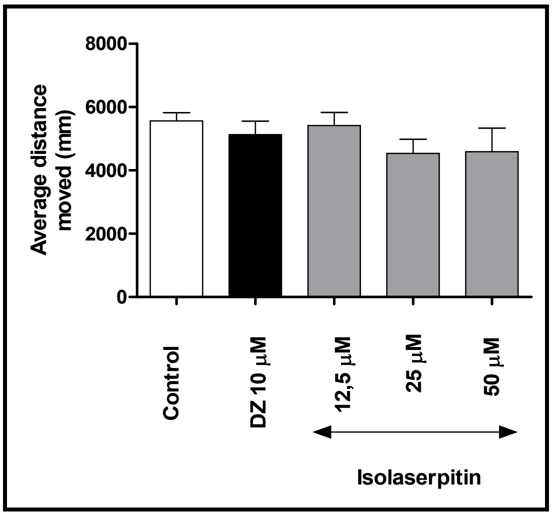

(**a**)

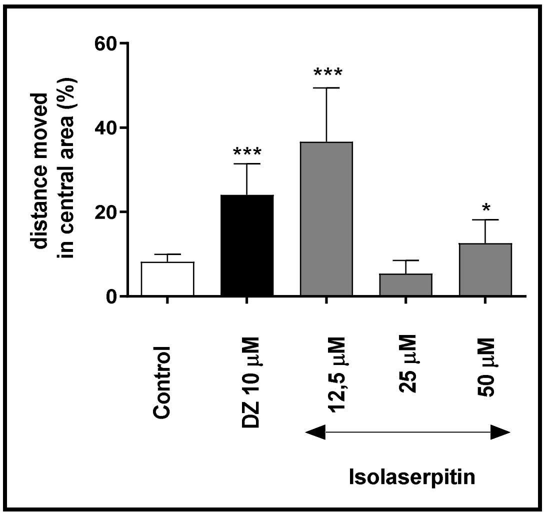

(**b**)

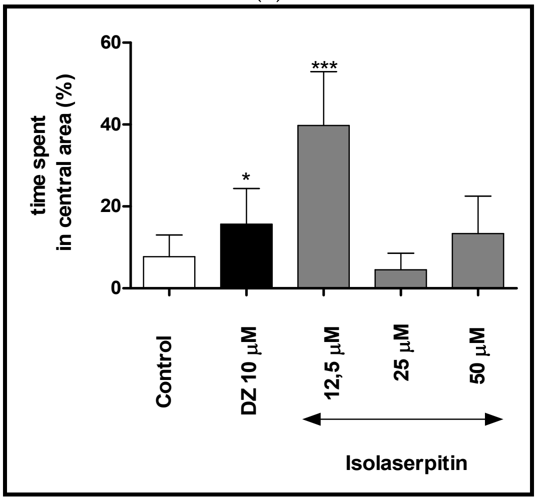

(**c**)

Figure S8 The effect of of isolaserpitin (12, 25, 50 μM)) and diazepam (10 μM) on locomotor activity during 40 min light phase. (**a**) Average distance (mm) moved by zebrafish larvae within each 1-min time bin; (**b**) the percentage of the distance moved in the central arena under the continuous illumination; (**c**) the percentage of the time spent in the central arena under the continuous illumination. Data are presented as mean ± SEM, n=32, **p<0.01, in comparison to control group, post hoc Tukey's test

C6. The influence of octanoyllomatin on spontaneous locomotor activity and thigmotaxis behaviour of the zebrafish larvae

The spontaneous locomotor activity was measured as average distance (in mm) moved in a minute during 40 minutes of continuous illumination. One-way ANOVA [F(4, 150)=0.3661; p=0.8307] revealed that octanyllomatin in all concentrations (9, 15, 30 μM) has not influenced the spontaneous locomotor activity **(Figure S9a)**.

One-way ANOVA showed statistically significant differences between groups concerning distance moved in the central arena ([F(4, 150)=9.095 p=0.0002], as well as time spent in the central arena [F(4, 150)=7.495, p=0.0004] after octanyllomatin (9, 15, 30 μM) incubation. Post-hoc test (Tukey's test) confirmed that as well diazepam (p<0.01) increased these parameters in comparison to DMSO-treated control group (**Figure S9b,c**).

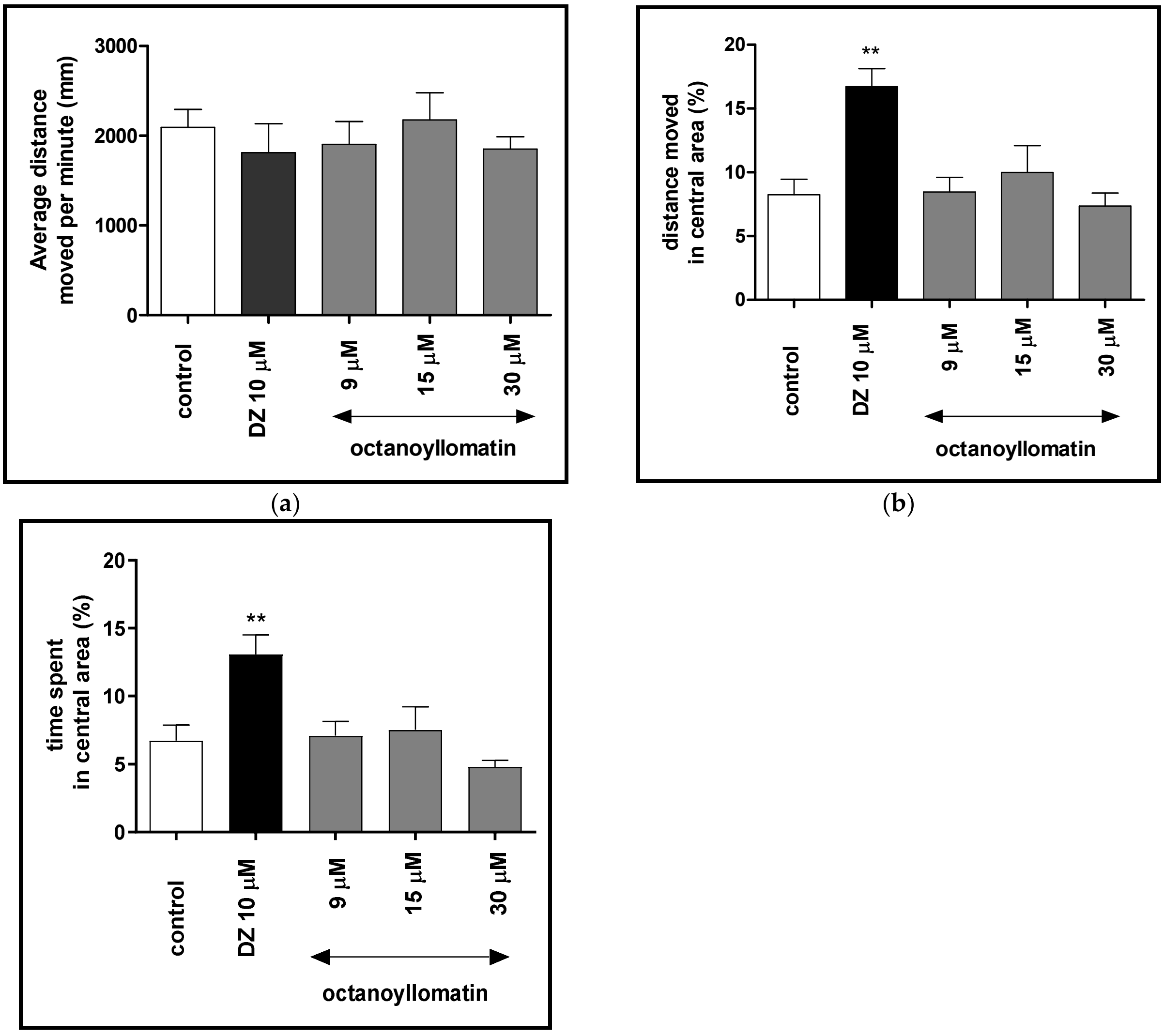

(c)

Figure S9. The effect of octanoyllomatin (9, 15, 30 μM) and diazepam (10 μM) on locomotor activity during 40 min light phase. (**a**) Average distance (mm) moved by zebrafish larvae within each 1-min time bin; (**b**) the percentage of the distance moved in the central arena under the continuous illumination; (**c**) the percentage of the time spent in the central arena under the continuous illumination. Data are presented as mean ± SEM, n=32, **p<0.01, in comparison to control group, post hoc Tukey's test.
